# Characterization and expression of AMP-forming Acetyl-CoA Synthetase from *Dunaliella tertiolecta* and its response to nitrogen starvation stress

**DOI:** 10.1038/srep23445

**Published:** 2016-03-30

**Authors:** Ming-Hua Liang, Xiao-Ying Qv, Hong-Hao Jin, Jian-Guo Jiang

**Affiliations:** 1 College of Food Science and Engineering, South China University of Technology, Guangzhou, 510640, China

## Abstract

AMP-forming acetyl-CoA synthetase (ACS) catalyzes the formation of acetyl-CoA. Here, a cDNA of ACS from *Dunaliella tertiolecta* (*DtACS*) was isolated using RACEs. The full-length *DtACS* cDNA (GenBank: KT692941) is 2,464 bp with a putative ORF of 2,184 bp, which encodes 727 amino acids with a predicted molecular weight of 79.72 kDa. *DtACS* has a close relationship with *Chlamydomonas reinhardtii* and *Volvox carteri f. nagariensis*. ACSs existing in Bacteria, Archaea and Eukaryota share ten conserved motifs (A1–A10) and three signature motifs (I–III) of the acyl-adenylate/thioester forming enzyme superfamily. DtACS was expressed in *E. coli* BL21 as Trx-His-tagged fusion protein (~100 kDa) and the enzymatic activity was detected. The recombinant DtACS was purified by HisTrap^TM^ HP affinity chromatography to obtain a specific activity of 52.873 U/mg with a yield of 56.26%, which approached the specific activity of ACS isolated from other eukaryotes. Kinetic analysis indicated that the *Km* of DtACS was 3.59 mM for potassium acetate, and the purified DtACS exhibited a temperature optimum of 37 °C and a pH optimum of 8.0. In addition, the expression levels of *DtACS* were increased after nitrogen starvation cultivation, indicating that ACS activity may be related to the lipid accumulation under nitrogen deficient condition.

Acetyl-CoA is an intermediate metabolite at the intersection of various anabolic and catabolic pathways, and its interconversion with acetate occurs by three distinct mechanisms^1^. One pathway consists of the acetate kinase (ACK, EC 2.7.2.1)/phosphotransacetylase (PTA, EC 2.3.1.8) enzymes, which catalyze acetate to acetyl-CoA via acetyl phosphate. Most anaerobic bacteria activate acetate to acetyl-CoA via ACK/PTA pathway. A second pathway of catalyzing acetate to acetyl-CoA is composed of ADP-forming acetyl-CoA synthetase (ADP-forming ACS, EC 6.2.1.13). It has been only existed in some archean halophytes and thermophiles, as well as in anaerobic protists^2^,^3^. A third route is composed of AMP-forming ACS (EC 6.2.1.1), and has a broader distribution and has been found in eubacteria, a few archaea, and eukaryotes^1^. In contrast to ACS, ACK and PTA function primarily in the catabolic direction, whereby acetate is excreted and ATP is synthesized. Hence, in bacteria, ACS is the preferred route of acetate assimilation. It seems that the role of ACS is more important in eukaryotes than in prokaryotes, since ACS is the only route for the activation of acetate to acetyl-CoA in eukaryotes.

AMP-forming ACS, which catalyzes the formation of acetyl-CoA from acetate, ATP and CoASH (acetate + ATP + CoASH → acetyl-CoA + AMP + PPi), is a member of the acyl-adenylate-forming enzyme superfamily that includes nonribosomal peptide synthetases, firefly luciferase, and acyl- and aryl-CoA synthetases^4^. ACS carries out an irreversible reaction via two enzymatic steps. The first step is to form acetyl-AMP by the reaction of acetate with ATP. Then acetyl-AMP reacts with CoASH to form acetyl-CoA releasing AMP. It was shown that the overexpression of ACS in *E. coli* caused significant reduction in acetate during glucose metabolism^5^. And the overexpression of *ACS2* in *Saccharomyces cerevisiae* also showed that the capacity of *S. cerevisiae* to synthesize acetyl-CoA from acetate was increased. It was presumed that increased ACS levels enhanced the formation of acetyl-CoA, which may increase the rate of fatty acid synthesis. Recently, the *E. coli ACS* gene was introduced into the marine microalga *Schizochytrium* sp. TIO1101, then the biomass and fatty acid proportion of ACS transformants were improved by 29.9% and 11.3%, respectively^6^. ACS overexpression could increase the flux toward acetyl-CoA from acetate, and therefore it was potentially important to enhance the production of fatty acid.

ACS has been cloned and expressed from several organisms, including Bacteria^7^,^8^, Archaea^9^,^10^, and Eukaryota^11^,^12^,^13^. All ACSs share several conserved sequence motifs and show high sequence identity^14^. The crystal structures of ACS from the bacterium *Salmonella enterica* and the yeast *S. cerevisiae* were reported^15^,^16^. Generally, molecular identification of ACS will provide the opportunity to learn more about the role of the corresponding gene product in lipid metabolism. To our knowledge, characterization of ACS from algae has not yet been reported. *Dunaliella tertiolecta*, one kind of green microalgae, contains large amount of lipid (36~42%) and has been reported to be a potential biodiesel feedstock^17^. In addition, *D. tertiolecta* is highly salt tolerant, simple to cultivate and not easy to be polluted, which make it possible to be large-scale outdoor cultivation^18^. In this study, the cDNA of *ACS* from *D. tertiolecta* (*DtACS*) was isolated by using reverse transcription-PCR (RT-PCR) and rapid amplification of cDNA ends (RACE) techniques. BLAST search displayed that the putative protein sequence exhibits the identities of 60~70% with the corresponding gene from other algae and plants, and ~50% with animal and fungi. Several conserved sequence motifs in DtACS were also found. Besides, the expression and purification of DtACS were carried out and the ACS activity was measured. And the transcription levels of *DtACS* were detected under nitrogen starvation stress.

## Materials and Methods

### Strains and Cultivation Conditions

*D. tertiolecta* cells were grown in a defined medium^19^ containing 1.5 mol/L NaCl at 26 °C under a 16/8 h dark/light cycle and were collected at the log phase or late log phase. *E.coli* DH5α was used as the host for the multiplication of plasmids.

### Cloning of the cDNA of *DtACS* from *D. tertiolecta*

The total RNA was prepared from 8 mL of *D. tertiolecta* cells grown at the late log phase with RNAiso plus reagent (Takara). The reverse transcription (RT) reaction was performed by the procedure: 42 °C, 60 min; 70 °C, 5 min, according to the RevertAid First Strand cDNA Synthesis Kit (Thermo Scientific). In order to clone the *DtACS* cDNA, two degenerated primers (5′-HTNGCNTGYKCNMGNATYGG -3′ and 5′-TCNGCNGTNCCRATDCKRTG-3′) were designed based on the two conserved amino acid regions (upstream L(M)ACA(S)RIG and downstream HRI(M)GTAE), Supplemental Fig. 1) from the ACS protein sequences of several species (*Chlamydomonas reinhardtii*, XM_001700178.1; *Volvox carteri f. nagariensis*, XM_002948417.1; *Bathycoccus prasinos*, XM_007509664.1; *Ostreococcus lucimarinus* CCE9901, XM_001416905.1; *Micromonas* sp. RCC299, XM_002506793.1; *Arabidopsis thaliana*, AF036618.1; *Arabidopsis thaliana*, NM_123046.3; *Solanum tuberosum*, X98506.1; *Medicago truncatula*, XM_003624510.1; *Ricinus communis*, XM_002512739.1; *Morus notabilis*, XM_010092639.1; *Theobroma cacao*, XM_007032042.1), then the expressed sequence tag (EST) of *DtACS* was acquired.

Then 5′-RACE-Ready cDNA was synthesized by a modified oligo (dT) primer, 5′ SMARTer II A Oligonucleotide primer and the SMARTScribe™ Reverse Transcriptase (a variant of MMLV RT) (BD Clontech). On the basis of the obtained EST of *DtACS*, two gene specific primers (5′-GTGTGAAGGGAGTGCCCGCAGCCT-3′ and 5′-GCCTTGCAGTCCTCCATGCGCCCT-3′) were designed to carry out the 5′ RACE-PCR. The following gradient PCR procedure was used: 95 °C, 3 min; 5 cycles of 94 °C, 30 s, 72 °C, 3 min; another 5 cycles of 94 °C, 30 s, 70 °C, 30 s, and 72 °C, 3 min; a final 20 cycles of 94 °C, 30 s, 68  °C, 30 s, and 72 °C, 3 min; at last, 72 °C, 5 min. For nested PCR of 5′-RACE, the following PCR procedure was used: 95 °C, 3 min; 25 cycles of 94 °C, 30 s, 68 °C, 30 s, and 72 °C, 3 min; at last, 72 °C, 5 min.

3′-RACE cDNA was synthesized using the RNA PCR Kit (AMV) Ver.3.0 (Takara) by Oligo dT-Adaptor Primer supplied; the first PCR of 3′-RACE was using a specific upstream primer (5′-TGGTGTGGAGCCTGTCATTCTGGA-3′) and Oligo dT-Adaptor Primer. For nested PCR of 3′-RACE, the primers were used as followed: another specific upstream primer (5′-TATGGCGACCACAAGCGGTATGAG-3′) and Adaptor Primer (5′-GTTTTCCCAGTCACGAC-3′). The PCR procedure was used as followed: 94 °C, 5 min; 30 cycles of 94 °C, 30 s, annealing temperature, 30 s, and 72 °C, 1 min/kb; 72 °C, 10 min.

All of the amplified fragments were cloned into pEASY-T1 vector (TransGen Biotech) and transformed into *E. coli* DH5α for multiplication, then sequenced before the further experiments.

### Sequence Analysis and Phylogenetic Construction

Sequence analysis was performed using BLAST (http://blast.ncbi.nlm.nih.gov/). Multiple alignments among similar enzymes were conducted using ClustalX 2.1. Physical and chemical features of DtACS were analyzed by ProtParam tool (http://expasy.org/tools/protparam.html). Conserved domains in DtACS were detected using the NCBI Conserved Domains Search (http://www.ncbi.nlm.nih.gov/Structure/cdd/wrpsb.cgi). Subcellular localization was predicted by PSORT prediction (http://psort.hgc.jp/form.html) and TargetP 1.1 (http://www.cbs.dtu.dk/services/TargetP/). Protein domains, families and functional sites were predicted by Prosite (http://prosite.expasy.org/). The protein secondary structures were predicted by Phyre2 (http://www.sbg.bio.ic.ac.uk/phyre2/html/page.cgi?id=index). The three-dimensional (3D) structure of DtACS was automatically predicted by 3D-JIGSAW (http://bmm.cancerresearchuk.org/~3djigsaw/) and the results were visualized by Raswin software 2.7.2.1. Phylogenetic tree was constructed using the Neighbor-Joining method with MEGA 5.

### Heterologous expression in *E. coli* and purification of the recombinant DtACS

The ORF of *DtACS* was amplified by PCR with the primers 5′-CTT**GAATTC**ATGAAGTGCAGCGCACT-3′ and 5′-ATC**CTCGAG**GTTCCTGGTTTCTATTA-3′. Then the PCR product was cloned into pET32a via two restriction sites (*Eco*RI and *Xho*I). The vector pET32a-ACS was transformed into *E. coli* BL21 (DE3). *E. coli* BL21 (DE3)/pET32a-ACS was grown in 10 mL LB medium overnight, and then the culture was transferred into 1 L LB medium containing ampicillin at 37 °C, 220 r/min until the optical density (OD) at 600 nm was 0.6. Then the temperature was adjusted to the induction temperature and the expression was induced by the addition of isopropyl β-D-1-thiogalactopyranoside (IPTG) for 12 h. The cells were harvested and resuspended in phosphate buffer solution (PBS, pH 7.4). Then the cell suspension was sonicated on ice at the intensity of 3 s burst and 8 s cooling period for 30 min with an ultrasonic cell disruptor. The lysate was centrifuged at 4 °C, 14,000 g for 15 min and the supernatant was applied to the HisTrap^TM^ HP column (GE healthcare) pre-equilibrated with the binding buffer (20 mM potassium phosphate (pH 7.4), 500 mM NaCl, and 40 mM imidazole). Then the column was washed with binding buffer, followed by washing buffer (20 mM potassium phosphate (pH 7.4), 500 mM NaCl, and 100 mM imidazole). The His-tagged protein was eluted with elution buffer (20 mM potassium phosphate (pH 7.4), 500 mM NaCl, and 500 mM imidazole). The purity of collected protein was detected by SDS-PAGE. The collected protein containing DtACS with high purity were dialyzed against 100 mM Tris-HCl buffer (pH 8.0) overnight at 4 °C to remove the imidazole, and subsequently concentrated at 3,000 g for 10 min at 4 °C by ultrafiltration using a 30 kDa cut-off concentrator (Millipore, USA) in the storing buffer (100 mM Tris-HCl, pH 8.0). Protein concentrations were determined by the Modified Bradford Protein Assay Kit (Sangon Biotech, China).

### Western blot analysis

The proteins separated by SDS-PAGE were transferred to polyvinylidene difluoride (PVDF) membrane (Millipore Co.). The membrane was incubated with horseradish peroxidase (HRP)-conjugated rabbit anti-His Tag antibody (Sigma) for 1 h at 37 °C. The reaction product was visualized by tetramethylbenzidine (TMB) (Tiangen) staining.

### Enzyme assays

The ACS activity was measured by a modified method of Brown *et al.*^20^. The reaction mixture (1 mL) contained 100 μmol of Tris-Cl (pH 8.0), 10 μmol of potassium acetate, 5 μmol of L-malate, 5 μmol of MgCl_2_, 0.1 μmol of CoA, 10 μmol of ATP, 5 μmol of NAD, 2.75 U of citrate synthase (CS), 150 U of malate dehydrogenase (MDH), and a certain amount of protein. The reaction was initiated by ATP and proceeded at 37 °C for 5 min. The formation of acetyl-CoA from acetate was determined by coupling the reaction to the rate of reduction of NAD by MDH and CS, and detected at 340 nm. Specific activity was defined as μmol NADH·min^−1^·mg^−1^ and was evaluated based on the following equation:


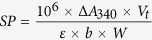


where SP is specific activity (U/mg), ∆A_340_ is the variation of the absorbance value at 340 nm compared with the control per minute; V_t_ (mL) was the total volume of the reaction; ε was the molar absorption coefficient of NADH, 6.22×10^3^ L·mol^−1^·cm^−1^; b was 1 cm optical path length of cuvette; w (mg), the content of protein.

### Kinetic analysis, pH and temperature dependence of DtACS

In order to the determination of Michaelis constant (*K*_*m*_) for two substrates, i.e. potassium acetate and sodium acetate, the final concentrations of these two substrate were determined as 2.5, 5, 10, 20, 40, 60 and 80 mM.

The pH dependence of DtACS was determined between pH 5.0 and pH 9.0 using either 2-(N-Morpholino) ethanesulfonic acid (MES) (pH 5.0 and 6.0) or Tris–HCl (pH 7.0, 8.0 and 9.0) (each 100 mM). The temperature dependence of the DtACS enzyme was determined between 15 °C and 50 °C, i.e. 15 °C, 25 °C, 37 °C and 50 °C.

### Nitrogen deficiency cultivation and quantitative real-time PCR (qRT-PCR) analysis

The optical density of the algal cultures was read at 630 nm (OD_630_) in a spectrophotometer. After 8 days of normal growth (OD_630_ = 07~0.8), the algal cells were harvested by centrifugation at 2,000 g for 5 min and resuspended in a fresh nitrogen-deficient medium containing no nitrogen source of the standard medium for a second phase of nitrogen deficiency cultivation for 7 days. In order to study the transcription level of *DtACS* under nitrogen-deficient condition, the total RNA was prepared from the algal samples (named ND 1d, ND 3d, ND 5d and ND 7d) cultivated in nitrogen-deficient medium for 1, 3, 5 and 7 days. And the algal samples (named Normal 0d, Normal 1d, Normal 3d, Normal 5d and Normal 7d) without nitrogen-deficient treatment continued to cultivate in a normal condition for 0, 1, 3, 5 and 7 days for comparison. qRT-PCR analysis was performed with a 7500 Real-Time PCR System (Applied Biosystems) using the PrimeScript RT Reagent Kit with gDNA Eraser (Takara) and the SYBR Green PCR Kit (Takara). The Primers for quantitative analysis are listed as followed: for *DtACS*, 5′-ATTCTGGACGACAAAGGCGT-3′ and 5′-TGGGTAGGGCCCAAAGTAGT-3′; and for endogenous gene, i.e. glyceraldehyde-3-phosphate dehydrogenase gene from *D. tertiolecta* (*DtGAPDH*), 5′-TGACTTCAAGGGCATGGACC-3′ and 5′-GTCGTACCAGGCAACAACCT-3′. Every sample was repeated three times to ensure statistical significance.

## Results

### Isolation and Characterization of the *DtACS* cDNA

**Figure 1:**
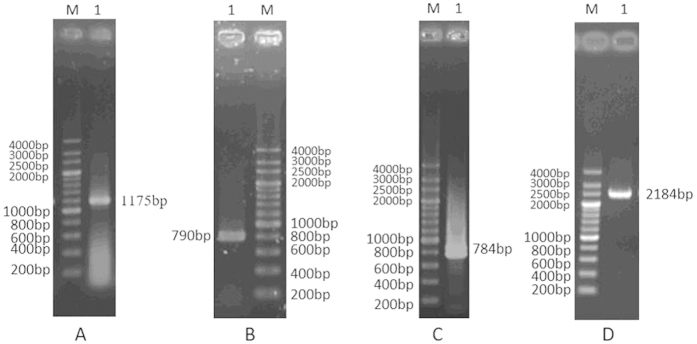
Isolation of *DtACS* cDNA. (**A**) *DtACS* EST; (**B**) 5′-end of the *DtACS* cDNA isolated by 5′ RACE; (**C**) 3′-end of the *DtACS* cDNA isolated by 3′ RACE; (**D**) Full-length *DtACS* ORF. Key: M, DNA ladder; 1, PCR products.

For the EST isolation of *DtACS*, according to the multiple sequences alignment result (Supplemental Fig. 1), a 1,175-bp fragment (Fig. 1) was isolated, and it showed high homology to *Chlamydomonas reinhardtii ACS* mRNA (XM_001700178.1) with 73% identity by BLAST, which suggested that the 1,175-bp fragment was from *ACS* of *D. tertiolecta*. Then two fragments corresponding to the 5′ and 3′ ends with 758 bp and 784 bp (Fig. 1) were isolated, respectively. It was found that the full-length cDNA of *DtACS* was 2,464 bp by sequence assembly and the coding sequence (CDS) was 2,184 bp flanked by a 6-bp 5′ untranslated region (URT) upstream of the start codon and a 274-bp 3′-UTR after the stop codon (Fig. 1). The 2,184-bp putative ORF encoded 727 amino acids, which showed 70% and 68% homology with those of *Volvox carteri f. nagariensis* and *C. reinhardtii*, respectively. It was showed that the molecular weight of DtACS was 79.72 kDa, and the isoelectric point was 6.70 by ProtParam tool.

### Sequence Analysis

**Figure 2:**
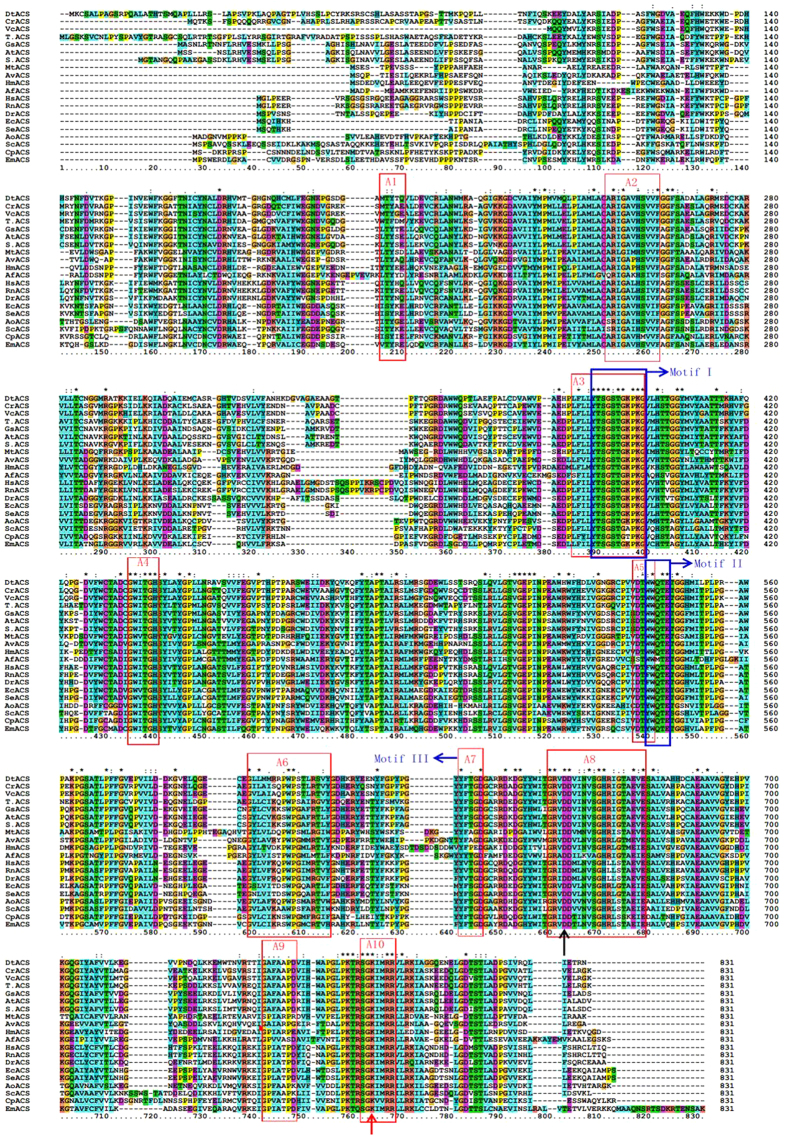
Multiple sequence alignment of amino acid sequences of ACS from all three domains of life: Bacteria, Archaea and Eukaryota. The alignment was generated with ClustalX 2.1. The ten conserved regions were highlighted by blue boxes. The three signature motifs (I–III) were highlighted in blue box. The red arrow indicated the lysine residue (K) essential for catalysis and posttranslational regulation. The black arrow indicated the aspartic acid residue (D) for the hinge of the N-terminus and the C-terminus. Name, NCBI accession numbers: for green algae: DtACS (in this study), *D. tertiolecta*, KT692941; CrACS, *Chlamydomonas reinhardtii*, XP_001700230.1; VcACS, *Volvox carteri f. nagariensis*, XP_002948463.1; T.ACS, *Tetraselmis* sp. GSL018, JAC81178.1; for Cyanobacteria: AvACS, *Anabaena variabilis* ATCC 29413, YP_321725.1; for archaebacteria: HmACS, *Haloarcula marismortui*, WP_011224682.1; AfACS, *Archaeoglobus fulgidus* DSM 4304, NP_069202.1; for higher plants: AtACS, *Arabidopsis thaliana*, NP_198504.1; GsACS, *Glycine soja*, KHN46374.1; S.ACS, *Saccharum* hybrid cultivar R570, AGT17010.1; for animals, HsACS, *Homo sapiens*, AAF75064.1; RnACS, *Rattus norvegicus*, NP_001101263.1; DrACS, *Danio rerio*, NP_001264046.1; for Bacteria: EcACS, *Escherichia coli*, WP_000078222.1; SeACS, *Salmonella enterica*, WP_031615207.1; MtACS, *Mycobacterium tuberculosis*, KHG66861.1; For fungi, AoACS, *Aspergillus oryzae* RIB40, XP_001820206.1; ScACS, *Saccharomyces cerevisiae* YJM789, EDN59709.1; for protists: CpACS, *Cryptosporidium parvum*, AAC47128.1; EmACS, *Eimeria maxima*, CDJ60264.1.

As shown in Fig. 2, DtACS showed a high identity to other ACSs from all three domains of life: Bacteria, Archaea and Eukaryota, and shared ten conserved motifs (A1–A10) and three signature motifs (I–III) of the acyl-adenylate/thioester forming enzyme superfamily^1^,^15^,^21^. It was shown that the A3, A5 and A7 motifs overlap with motifs I, II, and III, respectively^22^ (Fig. 2). The conserved hinge residue aspartic acid (Asp611 for DtACS) in A8 region bridged the N-terminal to the C-terminal of ACSs^15^. So DtACS contained a large N-terminal domain (1~611 amino-acid residues) and a small C-terminal domain (~110 amino-acid residues). The A10 conserved region had a common sequence of PXXXXGK, where X meant any amino acid. The lysine residue (Lys690 in DtACS) was reported to be crucial for catalysis of the adenylate-forming half-reaction^23^ and to be the site for sirtuin dependent posttranslational regulation of the ACSs of *Salmonella enterica* and *S. cerevisiae*^15^,^16^,^24^. The A3 region with gly-rich and Ser/Thr-rich sequence was supposed to play a part in directing the three phosphates of ATP before the first half-reaction^25^. The YGXTE residues in A5 conserved region could find the ATP binding pocket. The A3 and A10 regions were shown to take part in the first half reaction, and the A5 and A8 regions were involved in the second half reaction. In addition, a putative AMP-binding domain signature (at positions of 342~353 in DtACS amino acid sequence) has been predicted by Prosite tool, which was corresponding to A3 region.

PSORT indicates that the DtACS protein is in the plasma membrane; TargetP predicts that DtACS possesses a 64 amino acid transit peptide at its N-terminal, which directed DtACS to target to the chloroplast. The locations of DtACS should be determined by further study.

### Protein Structure Prediction

**Figure 3:**
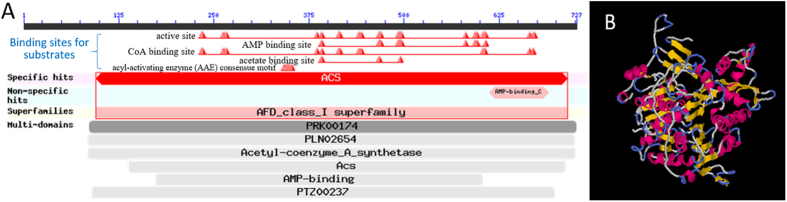
Conserved domains and 3D structure of the DtACS. (**A**) Conserved domains in DtACS detected by NCBI Conserved Domains Search. DtACS contained the binding sites for all substrates, i.e. Co A, AMP and acetate; (**B**) 3D model of the DtACS. Comparative modeling was performed using 3D-JIGSAW basing on homologues of known structures automatically. The structures were visualized using Raswin version 2.7.2.1. The α-helix and β-sheet regions of the putative protein are indicated with ribbons and arrows, respectively. The loop regions are also indicated in the schematics.

It was showed that the deduced protein DtACS contained the binding sites for all substrates, i.e. CoA, AMP and acetate by NCBI Conserved Domain Search (Fig. 3). The DtACS sequence showed high conservation in the CoA binding site, AMP binding site, acetate binding site, and active binding site when compared with ACSs from other species (Fig. 2).

The protein secondary structures were predicted by Phyre2. As shown in Supplemental Fig. 2, there were 36% α-helix region, 17% β-sheet region and 9% disordered region in DtACS. The 3D structure of the DtACS was modeled by 3D-JIGSAW, and was visualized by Raswin software 2.7.2.1 (Fig. 3).

### Phylogenetic Analysis

**Figure 4:**
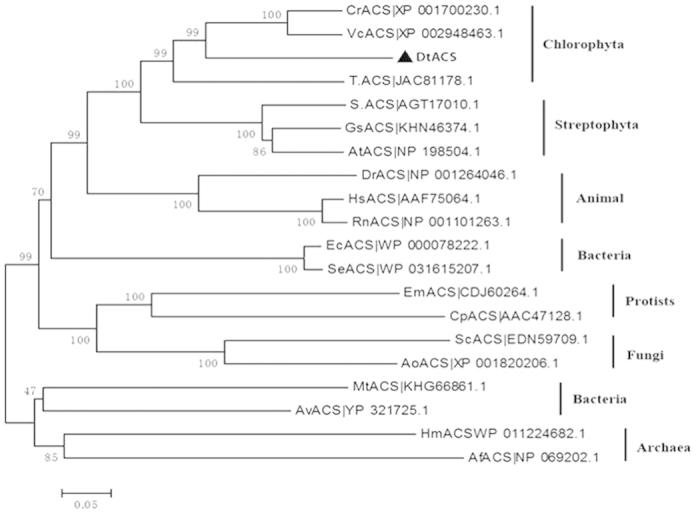
Phylogenetic relationships between different ACSs. Phylogenetic tree was constructed using neighbor-joining methods of the MEGA5 software package. Numbers associated with the branches were the neighbor-joining bootstrap values (n = 1000). The length of the branch expresses evolutionary distance with the scale being 0.05. The black triangle shows the position of the DtACS on the phylogenetic tree.

ACSs are existed in all three domains of life: Bacteria, Archaea and Eukaryota. The phylogenetic tree was shown in Fig. 4. It was shown that DtACS has a closer relationship with the ACSs of chlorophyte (*Chlamydomonas reinhardtii* and *Volvox carteri f. nagariensis*) and higher plants than those of other species.

### Protein expression and purification

The *DtACS* gene was successfully cloned into the expression vector pET32a and the recombinant DtACS with two 6·His-tag and a Trx-tag at the C-terminal (about 20 kDa) was expressed in *E. coli*. The recombinant DtACS seemed to be expressed mainly in the form of insoluble fraction. Then different temperatures (18 °C, 28 °C and 37 °C) and different concentrations of IPTG (0.3 mM, 0.6 mM and 0.9 mM) were tested to obtain maximum soluble DtACS protein. The optimal induction condition was 0.6 mM IPTG at 18 °C for 12 h when the *E. coli* cells were grown to OD_600_ = 0.6.

**Figure 5:**
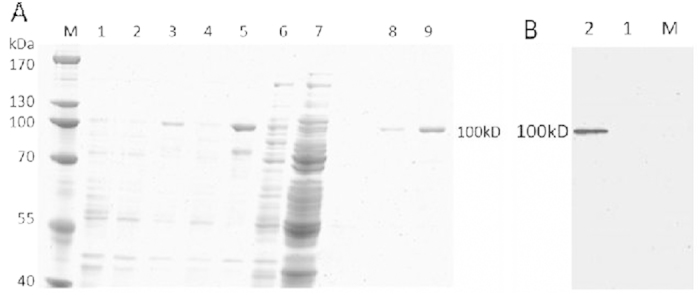
SDS-PAGE analysis and western blot analysis of the DtACS enzyme. (**A**) Purification of the recombinant DtACS from transformed *E. coli* BL21 as analyzed by SDS-PAGE. Lane M, molecular mass standards (Thermo); lanes 1, total protein of *E. coli* BL21/pET32a; lane 2, total protein of *E. coli* BL21/pET32a-ACS without IPTG induction; lane 3, total protein of *E. coli* BL21/pET32a-ACS after 12 h at 18 °C of IPTG induction; lane 4, crude extract (soluble fraction) of *E. coli* BL21/pET32a-ACS; lane 5, insoluble fraction of *E. coli* BL21/pET32a-ACS; lane 6, collected protein by binding buffer (40 mM imidazole); lane 7, collected protein by washing buffer (100 mM imidazole); lanes 8 and 9, collected protein (recombinant DtACS) by elution buffer (500 mM imidazole); (**B**) Western blot analysis of the DtACS enzyme. Lane M, protein marker; Lane 1, total protein of *E. coli* BL21/pET32a; lane 2, total protein of *E. coli* BL21/pET32a-ACS after 12 h at 18 °C of IPTG induction.

HisTrap^TM^ HP affinity chromatography was used to purify the soluble recombinant DtACS. After successive washing with washing buffer containing 40 mM imidazole and 100 mM imidazole, the protein was eluted with elution buffer containing 500 mM imidazole. It was showed that the DtACS protein fraction was eluted with 500 mM imidazole by SDS-PAGE analysis (Fig. 5), and a pure band corresponding to 100 kDa was in agreement with the theoretical mass of DtACS (79.72 kDa) with tags protein (about 20 kDa). This His-tagged protein was further examined by western blot using anti-His Tag antibody, and it was found that the recombinant DtACS could specifically bind to anti-His Tag antibody (Fig. 5).

### Enzymatic activity of DtACS

The ACS was purified 59.7-fold from cell extracts of *E. coli* BL21/pET32a-ACS by HisTrap^TM^ HP affinity chromatography to a specific activity of 52.873 U/mg with a yield of 56.26% (Table 1). This value was very close to the specific activity of ACS from other eukaryotes (30~60 U/mg)^11^. And it was higher than that of ACS from *Mycobacterium tuberculosis* (0.3~0.5 U/mg)^8^ and from the extremely halophilic archaeon *Haloarcula marismortui* (13 U/mg)^9^.

### Kinetic analysis, and effects of pH and temperature on the purified DtACS

**Figure 6:**
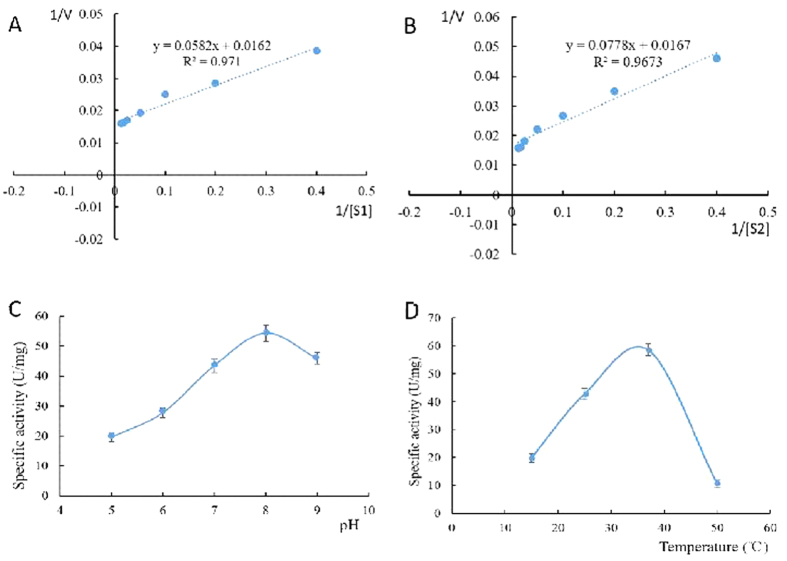
Characteristics of the purified ACS enzyme from *D. tertiolecta*. (**A**) Michaelis-Menten equation of DtACS for potassium acetate; (**B**) Michaelis-Menten equation of DtACS for sodium acetate; (**C**) Effect of pH on DtACS; (**D**) Effect of temperature on DtACS. Data were expressed as Mean ± SD of three independent experiments.

The dependence of the reaction rate on potassium acetate and sodium acetate concentrations followed Michaelis–Menten kinetics. From Fig. 6, the *Km* was 3.59 mM for potassium acetate and 4.66 mM for sodium acetate. It was indicated that the *Km* of ACS from *Sinorhizobium meliloti* was 5.8 mM for potassium acetate^26^. While it was reported that acetate was the preferred substrate for the ACS enzyme from *Mycobacterium tuberculosis* with a *K*_*m*_ of 1.2 mM^8^.

The effects of pH and temperature on ACS enzyme activity were determined. The optimal pH was found to be around pH 8.0 (Fig. 6). The DtACS enzyme was stable under neutral and alkaline conditions, while its activity decreased below pH 6.0. The optimal temperature for the DtACS enzyme was 37 °C (Fig. 6). The DtACS activity decreased rapidly at 15 °C or 50 °C.

### The results of qRT-PCR

**Figure 7:**
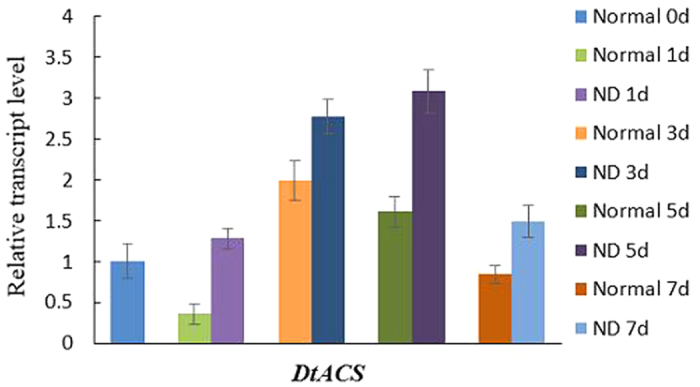
Transcription level of *DtACS* in *D. tertiolecta* in response to nitrogen deficiency analyzed by qRT-PCR. The data are expressed as Mean ± SD of three replicates.

The total RNA was prepared from all the algal samples mentioned in the Materials and Methods (for normal cultivation, Normal 0d, Normal 1d, Normal 3d, Normal 5d and Normal 7d; and for nitrogen-deficient cultivation, ND 1d, ND 3d, ND 5d and ND 7d), and the samples for normal cultivation were used as the control. Then the relative expression level of *DtACS* were analyzed by qRT-PCR (Fig. 7). It was showed that the transcription level of *DtACS* was increased after nitrogen-deficient cultivation, compared with the samples without nitrogen-deficient treatment and continued to be normal cultivation. After nitrogen-deficient cultivation by day 5, the expression level of *DtACS* came to be the maximum (3.082-fold higher than that of Normal 0d, and 1.472-fold of Normal 5d).

## Discussion

Acetate is a major metabolite in numerous organisms. And it can also be used as an alternative carbon source in microalgal heterotrophic or mixotrophic cultures. Research on microalgal growth of different species under heterotrophic or mixotrophic cultivation with acetate have been carried out. It was reported that 6-fold enhancement in biomass productivity and 5-fold enhancement in lipid content were obtained in *Chlorella pyrenoidosa* under mixotrophic cultivation with sodium acetate (10 g/L) compared with autotrophic cultivation^27^. It was also reported in *Chlamydomonas reinhardtii* that the greatest biomass (2.15 g/L) and fatty acid methyl esters (FAMEs) productivity (6.48 g/L/day) were observed for mixotrophic cultivation with acetate (10 g/L)^28^. The maximum lipid productivity in mixotrophic cultures with acetate in medium was 0.020 g/L/day in *Phaeodactylum tricornutum*^29^. As for *D. tertiolecta*, heterotrophic or mixotrophic cultivation with acetate has not yet been studied.

In eukaryotes, acetate can be converted into acetyl-CoA, a key intermediate in lipid synthesis, by acetyl-CoA synthetase (ACS). Recently, ACS overexpression has made process in the productions of fatty acids and fatty acid ethyl esters (FAEEs)^6^,^30^. FAEEs are produced from ethanol and acyl-CoA. And acyl-CoA are produced from large amounts of NADPH and acetyl-CoA. It was reported that *ADH2* encoding alcohol dehydrogenase and *ALD6* encoding acetaldehyde dehydrogenase were overexpressed together with the heterologous gene encoding ACS in *Saccharomyces cerevisiae*, resulted in a final FAEE yield of 408 ± 270 μg/g dry cell weight, a 3-fold improvement compared with the reference strain^30^.

ACS have been identified and purified from various species, such as the extremely halophilic archaeon *Haloarcula marismortui*^9^, the hyperthermophilic crenarchaeon *Pyrobaculum aerophilum*^10^, *Mycobacterium tuberculosis*^8^, *Saccharomyces Cerevisiae*^12^, *Phycomyces blakesleeanus*^31^, *Spinacia oleracea* L. 32 and human^13^. To our knowledge, few algal ACS has been reported. Here, the cDNA of *ACS* from *D. tertiolecta*, a haloduric green algae, was isolated, and the DtACS protein was expressed and purified in *E. coli*. DtACS showed high conservation with other ACSs from all three domains of life: eubacteria, archaebacteria, and eukaryotes (Fig. 2) and had ten conserved motifs (A1–A10) and three signature motifs (I–III) in common. DtACS had a closer relationship with the ACSs from chlorophyte (*Chlamydomonas reinhardtii* and *Volvox carteri f. nagariensis*) and higher plants than those of other species (Fig. 4). Enzyme activity of DtACS was also detected. The DtACS was purified with a specific activity of 52.873 U/mg with a yield of 56.26% (Table 1). This value was very close to the specific activity of ACS from other eukaryotes (30~60 U/mg)^11^. And it was reported that the specific activity of ACS was 0.3~0.5 U/mg from *Mycobacterium tuberculosis*^8^, and 13 U/mg from the extremely halophilic archaeon *Haloarcula marismortui*^9^. DtACS has a higher affinity with potassium acetate than with sodium acetate (Fig. 6). The optimal pH for the DtACS enzyme was found to be around pH 8.0 (Fig. 6), and the optimal temperature was 37 °C (Fig. 6).

Analyzed by qRT-PCR (Fig. 7), the transcription levels of *DtACS* were increased after nitrogen-deficient cultivation, compared with the corresponding normal samples. It was mentioned that in many cases the cultivation of microalgae under stress conditions (such as nutrient starvation, high salinity, high temperature etc.) could enhance significant accumulation of lipids^33^. It was reported that lipid contents were increased in *D. tertiolecta* under nitrogen deficiency condition, and cells cultivated in nitrogen-deficient medium accumulated largest amounts of lipid by days 3 to 5 17. In this study, it was found that the transcription levels of *DtACS* were increased under nitrogen-deficient cultivation, and the highest transcription level of *DtACS* happened at the nitrogen-deficient treatment by day 5. It was suggested that ACS activity may be related to the lipid accumulation in *D. tertiolecta*. In our another manuscript, we isolated another two genes (*DtACLA* and *DtACLB*, encoding two subunits of ATP-citrate lyase) involved in the formation of acetyl-CoA. It was found that the transcription levels of *DtACLA* and *DtACLB* after nitrogen deficiency were also increased. It seemed that the genes involved in the formation of acetyl-CoA were up-regulated in response to nitrogen starvation stress.

Taken together, the cDNA of *ACS* from *D. tertiolecta* has been isolated, and the DtACS protein has been purified. It has been identified that DtACS showed enzymatic activity. It was indicated that DtACS activity may be related to lipid accumulation under nitrogen-deficient cultivation of *D. tertiolecta* analyzed by qRT-PCR. In addition, heterotrophic or mixotrophic cultivation with acetate in *D. tertiolecta* can be conducted for further study.

## Additional Information

**How to cite this article**: Liang, M.-H. *et al.* Characterization and expression of AMP-forming Acetyl-CoA Synthetase from *Dunaliella tertiolecta* and its response to nitrogen starvation stress. *Sci. Rep.* **6**, 23445; doi: 10.1038/srep23445 (2016).

## Supplementary Material

Supplementary Information

## Figures and Tables

**Table 1:** Purification of ACS from *D. tertiolecta*.

Purification step	Protein(mg)	Activity (U)	Specific activity(U/mg)	Yield (%)	Purification(-fold)
Crude extract	305.200	270.102	0.885	100	1.0
After purification	3.717	211.575	56.921	78.33	64.3
After ultrafiltration	2.874	151.957	52.873	56.26	59.7

Enzyme activity was measured at 37 °C for 5 min as described in Materials and methods. 1 U = 1 μmol NADH·min^−1^.
