# Supplementary material for: Characterization and expression of AMP-forming Acetyl-CoA Synthetase from Dunaliella tertiolecta and its response to nitrogen starvation stress
Source: Sci Rep. 2016 Mar 30;6:23445. doi: 10.1038/srep23445 (PMC4812251; doi:10.1038/srep23445)
Supplement: Supplementary Information [file srep23445-s1.pdf]

Characterization and expression of AMP-forming Acetyl-CoA Synthetase from *Dunaliella tertiolecta* and its response to nitrogen starvation stress

Ming-Hua Liang, Xiao-Ying Qv, Hong-Hao Jin, Jian-Guo Jiang *

College of Food Science and Engineering, South China University of Technology, Guangzhou, 510640, China

*Author (Jian-Guo Jiang) for correspondence (*e-mail*: jgjiang@scut.edu.cn; phone: +86-20-87113849; fax: +86-20-87113849).

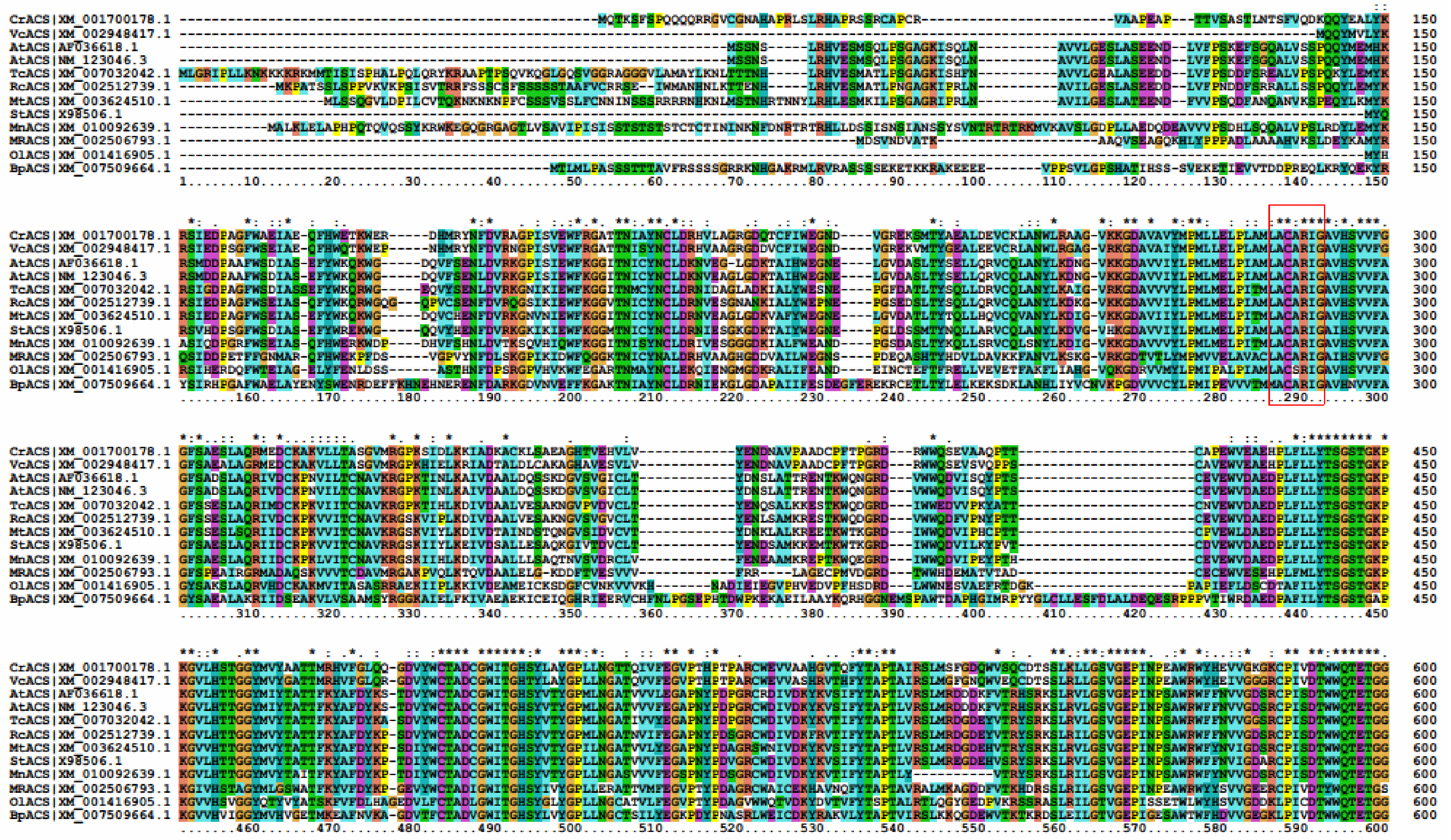
CrACS|XM_001700178.1
VcACS|XM_002948417.1
AtACS|AF036618.1
AtACS|NM_123046.3
TcACS|XM_007032042.1
RcACS|XM_002512739.1
MtACS|XM_003624510.1
StACS|X98506.1
MnACS|XM_010092639.1
MRACS|XM_002506793.1
OlACS|XM_001416905.1
BpACS|XM_007509664.1
150
300
450
600

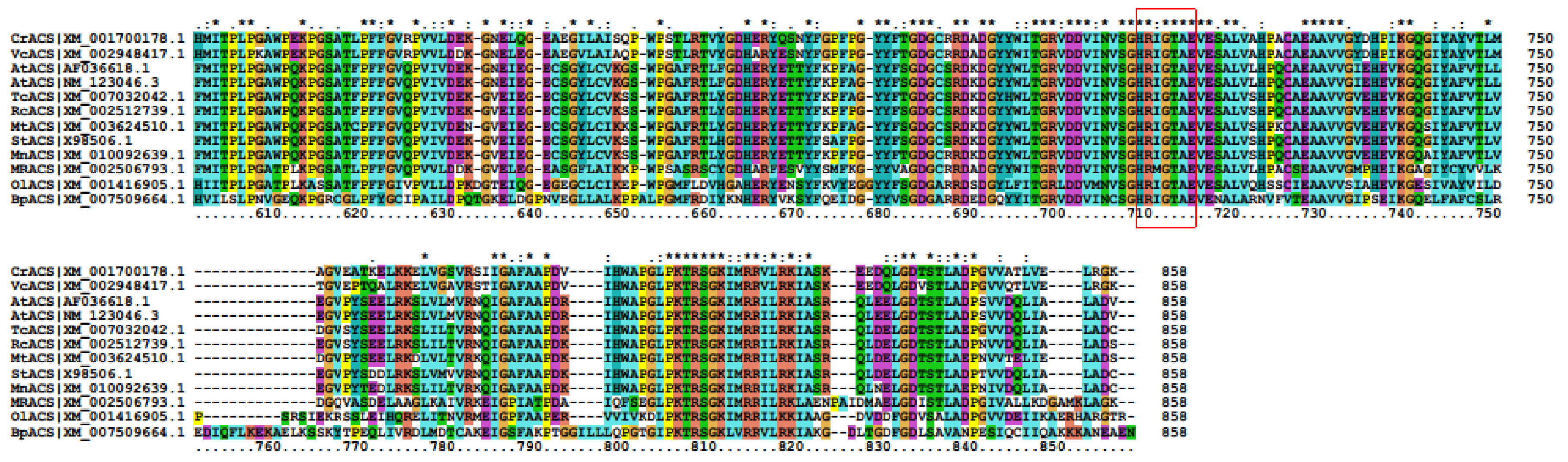

Supplemental Fig. S1 Multiple sequences alignment of ACSs from other species.

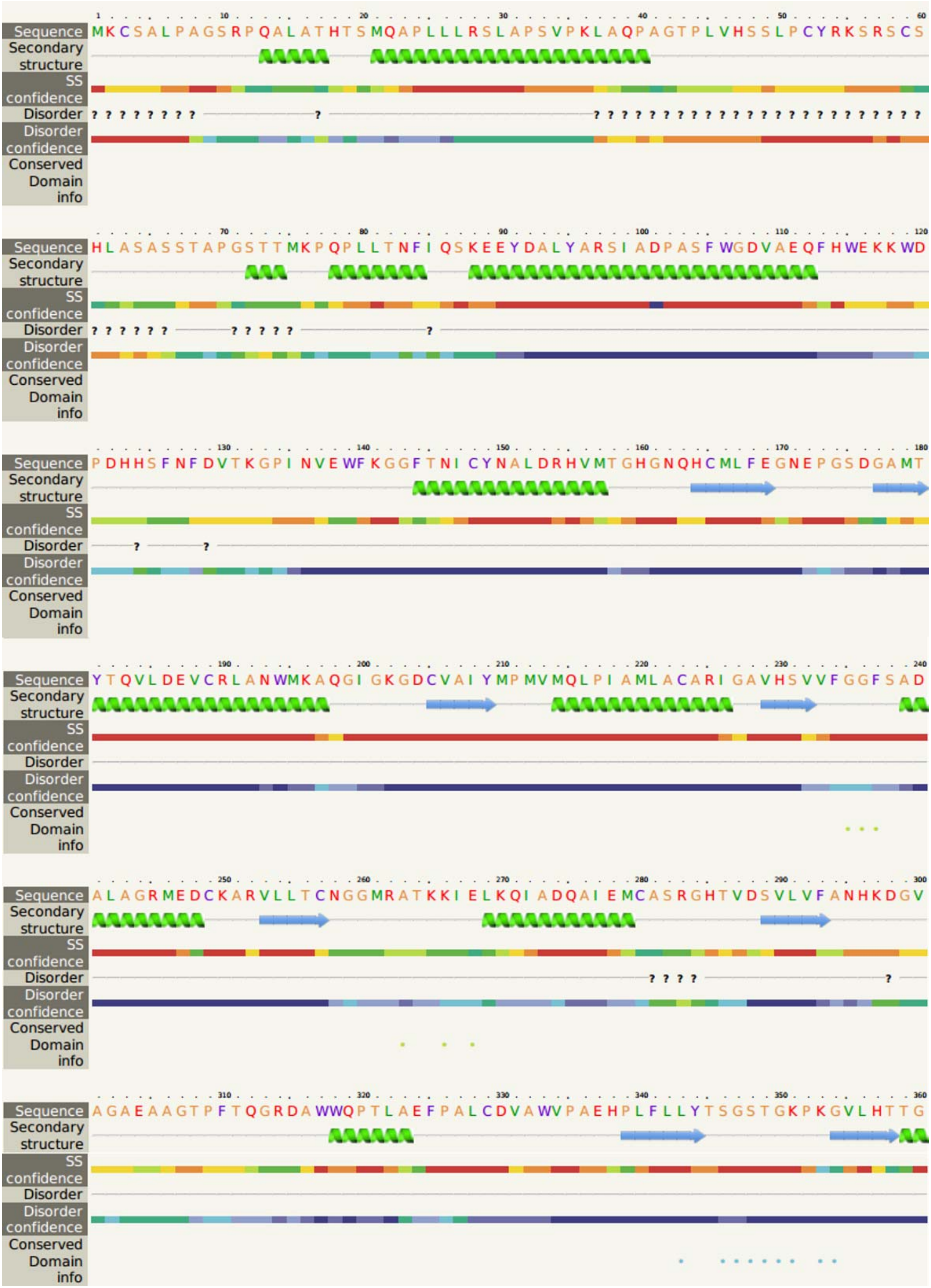

Sequence
Secondary structure
SS confidence
Disorder
Disorder confidence
Conserved Domain info
1 10 20 30 40 50 60
MKCSALPAGSRPQALATHTSMQAPLLLRSLAPSVPKLAQPAGTPLVHSSLPCYRKSRSCS
70 80 90 100 110 120
HLASASSTAPGSTTMKPQPLLTNFIQSKEEYDALYARSIADPASFWGDVAEQFHWEKKWD
130 140 150 160 170 180
PDHHSFNFDVTKGPINVEWFKGGFTNICYNALDRHVMTGHGNQHCMLFEGNEPGSDGAMT
190 200 210 220 230 240
YTQVLDEVCRLANWMKAQGIGKGDCVAIYMPMVMQLPIAMLACARIGAVHSVVFGGFSAD
250 260 270 280 290 300
ALAGRMEDCKARVLLTCNGGMRATKKIELKQIADQAIEMCASRGHTVDSVLVFANHKDGV
310 320 330 340 350 360
AGAEAAGTPFTQGRDAWWQPTLAEFPALCDVAWVPAEHPLFLLYTSGSTGKPKGVLHTTG

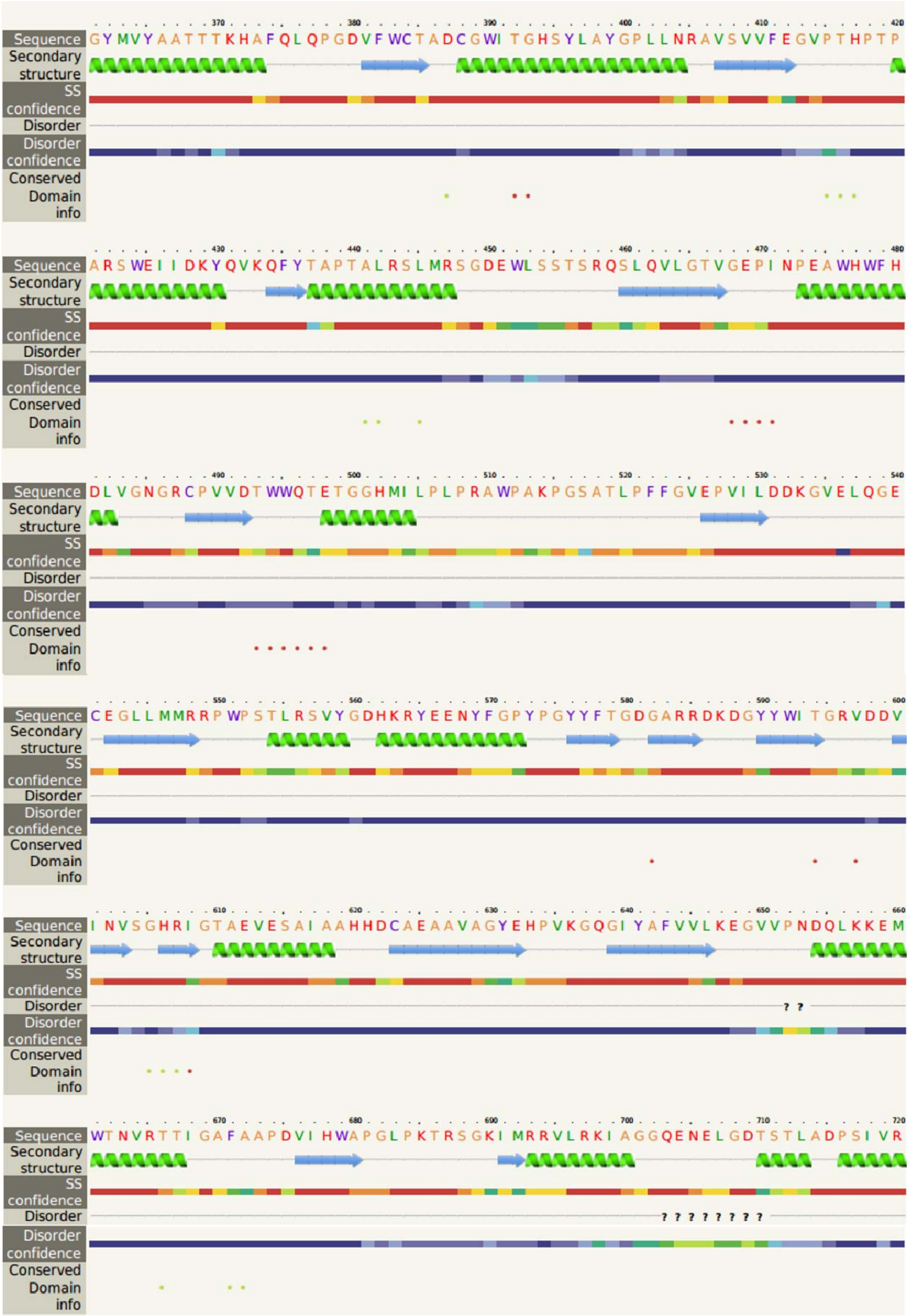

370 380 390 400 410 420
Sequence GYMVYAATTTKHAFQLQPGDVFWCTADCGWITGHSYLAYGPLLNRAVSVVFEGVPTHPTP
430 440 450 460 470 480
Sequence ARSWEIIDKYQVKQFYTAPTALRSLMRSGDEWLSSTSRQSLQVLGTVGEPINPEAWHWFH
490 500 510 520 530 540
Sequence DLVGNGRCPVVDTWWQTETGGHMILPLPRAWPAKPGSATLPFFGVEPVILDDKGVELQGE
550 560 570 580 590 600
Sequence CEGLLMMRRPWPSTLRSVYGDHKRYEENYFGPYPGYYFTGDGARRDKDGYYWITGRVDDV
610 620 630 640 650 660
Sequence INVSGHRIGTAEVESAIAAHHDCAEAAVAGYEHPVKGQGIYAFVVLKEGVVPNDQLKKEM
670 680 690 700 710 720
Sequence WTNVRTTIGAFAAPDVIHWAPGLPKTRSGKIMRRVLRKIAGGQENELGDTSTLADPSIVR
Secondary structure
SS confidence
Disorder
Disorder confidence
Conserved Domain info

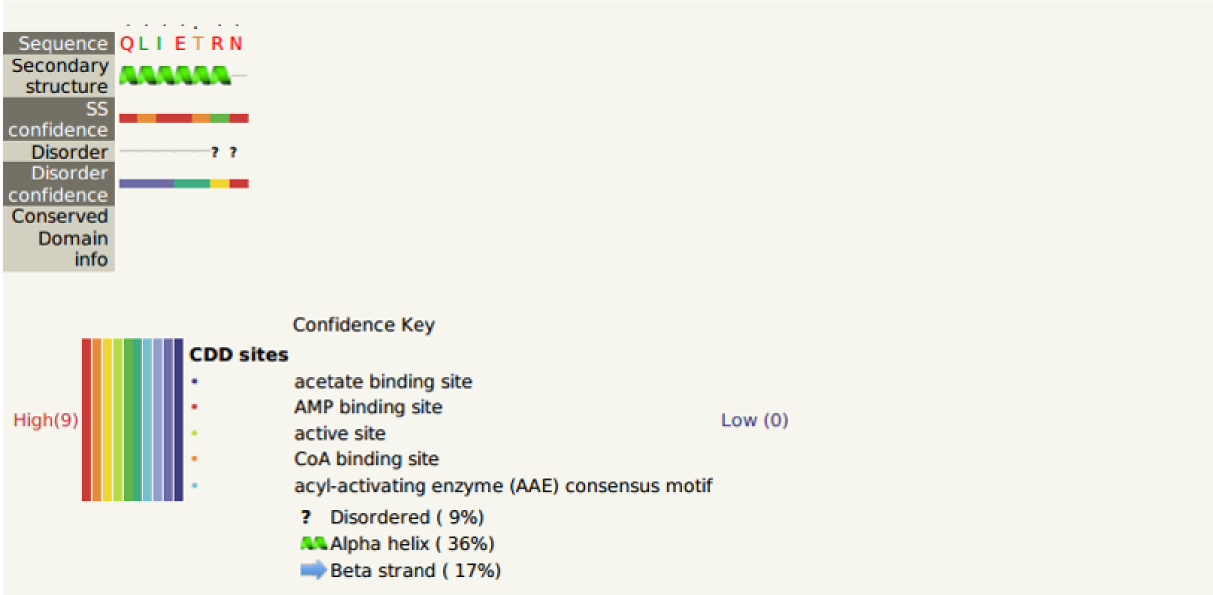

Supplemental Fig. S2 The protein secondary structure of DtACS predicted by Phyre2.
